# Supplementary material for: Development of Roselle (Hibiscus sabdariffa L.) Transcriptome-Based Simple Sequence Repeat Markers and Their Application in Roselle
Source: Plants (Basel). 2024 Dec 16;13(24):3517. doi: 10.3390/plants13243517 (PMC11679260; doi:10.3390/plants13243517)
Supplement: Supplementary file 1 [file plants-13-03517-s001.zip › Supplementary Table S1.pdf]

Supplementary Table S1. The information of 32 pairs of SSR primers with polymorphism.

SSR Code	SSR type	Repeat type	Primer sequence（5′-3′）	Size of amplifying sequence (bp)
SSR 8	p2	(CT)11	ACTTAGCTCAAGCTGGCGAC	184
SSR 10	p2	(TC)13	GGAAAATAGACAGATTCATCCAAA	205
SSR 15	p2	(AG)13	GAAGGAAAGTGTTGAAACCCA	164
SSR 17	p3	(CAG)8	CAGCAAGAACAGGAACCACA	171
SSR 36	p2	(TC)10	TCCTTTTTGACTTGGGGATG	258
SSR 39	p2	(CT)10	GAAGCAGCTCCAAACTCACC	153
SSR 42	p3	(TCC)7	TGAACCACTCTTCCCATCCT	271
SSR 44	p2	(TC)10	CCGTTTTCAATTTCATCGGT	251
SSR 45	p3	(GCT)7	CGGAAAAAGAAGTGCCTGTC	171
SSR 49	p2	(AG)12	CATAAAATCCATCCGCTTTCA	240
SSR 50	p2	(TC)18	TCTTCAACGGCCCTTTTCTA	236
SSR 55	p3	(CCA)7	CCAACATCATCAGCACCATC	268
SSR 56	p4	(CAGG)5	ATCACAGTCGAGTGGGAACC	200
SSR 58	p2	(AC)15	TCGCTGTAATTTGAAGTTTTTCAG	249
SSR 59	p3	(CTG)7	CCTCTCTTTTGCCCTCCTCT	226
SSR 60	p4	(TGCC)6	ATGGTGGATCTACCCACAGG	213
SSR 62	p3	(CTC)9	ATATTAGTTGGCCGGAGGCT	195
SSR 66	p3	(GGT)7	TTGGAAGGGGTAGAGGAGGT	177
SSR 74	p3	(CTG)9	GCGGAGAGAGAAGAACCCTT	227
SSR 76	p2	(CT)14	TGCAGCTTATCTGACACCGT	158
SSR 77	p2	(AG)15	CGAGAACTAAAAGTTGAAACTGGA	245
SSR 78	p4	(ATGG)5	GAAAACAAAAACAAGGGGCA	274
SSR 80	p2	(GA)20	TGAAGCCATGAGAAATGCTG	278
SSR 82	p3	(CAC)7	ACCGCCTTTCAAGTCCTGTA	195
SSR 83	p2	(AG)12	GACACCAGGCACTGCTCTTA	153
SSR 84	p2	(AG)15	GTCCTGACGTGGATCGATTT	179
SSR 87	p2	(GA)14	CGCCATTGTTAAAGCTGGA	235
SSR 89	p2	(CT)12	TCTTGCTGTCAGTAATCTGGGA	188
SSR 94	p2	(CT)10	TTTTTGCATCCCTATCGACC	182
SSR 98	p4	(TGGA)5	AAAGGCACAAGAGAGGAACG	251
SSR 99	p2	(TG)10	CCTGCAACTGTTCTTCCTCC	196
SSR 100	p2	(GA)10	GTCAAGGGCCTGAAACACAT	253
